# Comparative use of a polarized light compass for twilight and moonlight navigation in diurnal and nocturnal bull ants

**DOI:** 10.1098/rsos.250975

**Published:** 2025-11-12

**Authors:** Cody A. Freas, Ken Cheng

**Affiliations:** ^1^ School of Natural Sciences, Macquarie University, North Ryde, New South Wales, Australia; ^2^ Research Center on Animal Cognition (CRCA), Center for Integrative Biology (CBI), CNRS, Toulouse, France

**Keywords:** ant navigation, cue detection, lunar navigation, crepuscular, polarized moonlight

## Abstract

We present evidence that the faint polarized moonlight pattern of the sky can be used for navigation in a diurnal animal, the bull ant *Myrmecia tarsata*. This comes despite this species lacking the highly refined low-light visual specializations of nocturnal bull ants. Celestial bodies can provide animals with directional information, yet direct observation can often be occluded. Positional information of solar and lunar cues can be estimated via their polarized light pattern, present across the sky. The sun’s polarization pattern is widely used in animals, and a similar, yet much fainter, pattern is produced by the moon. However, given the inherent variability of the moon, it is unknown how widespread moonlight use is in navigating animals. Here, *M. tarsata*, which forages throughout the day, returning home at sunset, uses both solar and lunar polarized light to navigate. We compare this to the closely related nocturnal sympatric *Myrmecia midas* navigating under identical conditions. Both species clearly use solar and lunar polarized light patterns to navigate, but *M. tarsata* showed degraded performance under polarized moonlight as a function of lunar phase, decreasing performance as illumination decreased. *Myrmecia midas*, in contrast, exhibited impressive attendance to lunar polarization patterns throughout the lunar month.

## 1. Background

Polarized light describes light waves that oscillate in a directionally predictable fashion. When these waves oscillate along a single plane, they are defined as linearly polarized. Polarization can arise naturally, with both sunlight and moonlight becoming polarized as a by-product of passing through the Earth’s upper atmosphere [1,2], producing a characteristic pattern across the sky. This pattern is organized in concentric circles around the sun, with the angle of polarization (e-vector) oriented tangentially to these circles. The degree of polarization, or the proportion of light that is polarized, increases with angular distance from the celestial body and reaches its maximum about 90° away. Thus, at sunrise, sunset and during twilight, the overhead sky exhibits a strong, linear polarization pattern that is nearly perpendicular to the position of the sun and roughly parallel to the north–south axis [3–6]. The polarization pattern of the sun is present across the sky throughout the day and into twilight (when the sun is below the horizon between 0° and –18°) and is a useful compass cue for animal orientation and homing [4–12]. Many animals navigate or hold a heading direction via the azimuthal position of the sun or moon, yet these can often be obscured by cloud cover or local vegetation [11,13–18]. Thus, the polarization pattern provides a predictable proxy for where the sun is positioned even when they are not directly visible [1].

When the sun is near the horizon, the concentric pattern of polarization, or angle of polarization, collapses into a single axis roughly aligned north to south (e-vector) across the sky. This results in a directionally ambiguous pattern along the 180° axis, making it difficult to distinguish between the sun and anti-sun directions. Such 180° ambiguity can disorient animal navigators relying solely on polarized light. However, many insect navigators mitigate this ambiguity by integrating multiple celestial and terrestrial cues, dynamically weighting the polarization pattern with other inputs such as the visual panorama and spectral gradients to resolve directional uncertainty [7,19–22].

Insects commonly detect and use the sky’s polarization pattern via polarization-sensitive photoreceptors in the dorsal rim area of the compound eye [4,12,23–28], as well as their ocelli [29–34]. Much of what we know about polarized-light navigation focuses on diurnal animals utilizing solar light polarization, yet the moon also produces a similar yet much dimmer pattern radiating from its position, by reflecting sunlight off its surface [35]. Multiple species of insects and other arthropods, including ants and bees, are known to track the moon’s observable position as part of their celestial compass [15,16,36–38], yet navigation via lunar polarized light remains understudied. Given that the degree of polarization is significantly lower at night than during the day [39], this pattern has been shown to be observable by nocturnal animals which possess specialized eye physiologies for low light [4,36,39–42]. Two species of nocturnal dung beetles (*Scarabaeus satyrus* and *Scarabaeus zambesianus*) can attend to polarized moonlight for heading maintenance while they roll dung balls in a straight line away from a central dung pile [4,13,36,39,42]. This short-term use of lunar polarization to maintain a fixed bearing represents orientation. This is distinct from navigation, which is goal-directed, such as insects maintaining a path integration-derived estimate (vector) of their nest location. The large-eyed ants of the *Myrmecia* genus include species that possess highly attuned systems for navigating at low light [43–47]. Among them, the nocturnal bull ant *Myrmecia midas* is remarkable for its ability to use the lunar polarization pattern as a compass cue to update its path integrator during nocturnal foraging, just as diurnal ants use the sun’s pattern as part of their daytime celestial compass [48,49]. Moreover, *M. midas* overnight navigation appears heavily dependent on the moon, with foragers maintaining a time-compensated lunar compass, adjusting for the moon’s changing azimuth across the night to maintain an accurate homeward vector [38]. This nocturnal path integrator using the external lunar-compass references enables goal-directed navigation throughout the night. Beyond these species, it has been hypothesized that this cue may be broadly used across nocturnal insect groups [50–53].

Given that the moon creates such a dim version of the polarization pattern formed around the sun, an initial prediction might be that visual specializations for low-light sensitivity, particularly in the dorsal rim area, would be needed to reliably detect and attend to this pattern in order to orient and navigate to goals. Yet evidence suggests detection may be more widespread: the diurnal dung beetle *Scarabaeus* (*Kheper*) *lamarcki* also attends to moonlight polarization patterns during their movement [42], suggesting that moonlight may be both detectable and a reliable compass cue for insects at night regardless of their temporal activity niche. Though importantly, *S. lamarcki* does exhibit degraded orientation performance compared to their nocturnal counterparts, especially under crescent-moon-derived polarization patterns [39,42], hinting that lunar cues may not be usable to diurnal animals throughout the lunar cycle. Unlike the sun, the lunar polarization pattern varies with its phase: as the illuminated portion of the lunar surface decreases, the amount of reflected polarized light scattered into the Earth’s sky diminishes, resulting in a progressively weaker polarization pattern.

When exploring the possibility of widespread use of polarized moonlight in navigating insects, diurnal/crepuscular species that possess some level of visual specialization for dim light would be prime initial candidates for study [46,51,54–56]. The diurnal/crepuscular bull ant *Myrmecia tarsata* exhibits many of these attributes. This species forages mainly during the day, yet its afternoon inbound foraging trip coincides with sunset and persists into the evening twilight [51]. Thus, foragers likely can navigate at least somewhat effectively in the declining light levels of twilight, relying on the solar polarization pattern to inform their celestial compass, akin to other diurnal/crepuscular ants [8].

**Figure 1.**
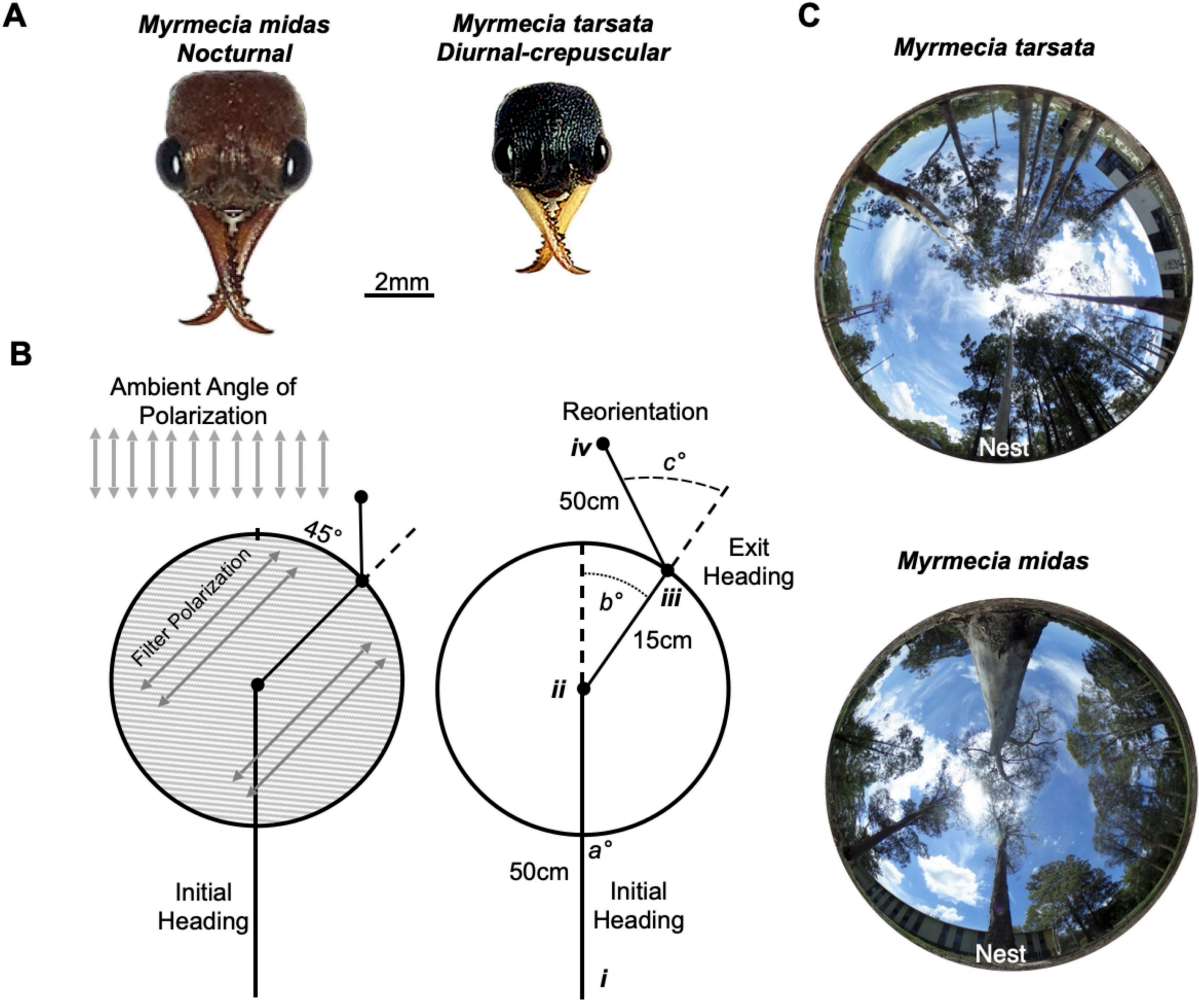
Diagram of the changes to the overhead polarization pattern using the linear polarized filter during testing in *M. tarsata* and *M. midas*. (A) Dorsal images of a forager’s head of each species. (B) The ambient lunar polarization pattern, or e-vector, was altered by ±45°. Foragers’ initial heading directions, from release (*i*) to the site where the filter was placed over each forager (*ii*). Exit headings were calculated from the centre of the filter to (*ii*) the forager’s exit at the filter edge (*iii*). Shifts under the filter (*b*°) were calculated by taking the difference from the initial heading and the exit heading. Reorientations were measured from the filter edge (*iii*) to the forager’s heading 50 cm post-filter exit (*iv*). Reorientation headings (*c*°) were calculated from the angular difference from the exit headings to reorientation heading direction. (C) Images of the sky and canopy cover at the two nests. Photos were taken at the on-route testing area for both nests.

In the current study, we compared the use of the overhead polarized-light pattern (both solar and lunar) in two sympatric bull ant species, the diurnal/crepuscular *M. tarsata* and the nocturnal *M. midas*. We characterized both species’ ability to orient under solar polarized-light cues, during evening or morning twilight (corresponding with each species’ natural inbound navigation periods), by rotating a linear polarizing filter over them as they navigated to their nest (figure 1). Additionally, we explored both species’ ability to detect and attend to polarized moonlight during a full moon, when lunar cues are brightest, as well as across the lunar cycle, during quarter-moon and crescent-moon nights, when smaller portions of the moon’s surface reflect sunlight, producing a weaker lunar polarized light pattern. *Myrmecia tarsata* naturally returns to its nest during evening twilight, suggesting it likely naturally uses the solar polarization pattern during this period to navigate, like other twilight ant navigators [5–8]. As this species is not typically active at night, it is unknown if it can detect and attend to lunar polarized light to navigate. For a comparative perspective, we tested *M. midas* foragers during similar conditions, as it is a well-studied nocturnal bull ant which we know uses both the overhead solar and lunar polarized-light patterns [7,48], even under crescent moons when the lunar polarization pattern is at its faintest.

## 2. Methods

### 2.1. Study site

Experiments were conducted from August 2024 to January 2025 on one *M. tarsata* nest and one *M. midas* nest in Sydney, Australia (33°46 11 S, 151°06 40 E). Both nests were located within eucalyptus tree stands with largely barren understories (figure 1) and separated by 440 m. *Myrmecia tarsata* is a largely diurnal forager, exhibiting two distinct bouts of foraging traffic, one in the morning with foragers returning before midday and one in the afternoon with foragers returning around sunset and into evening twilight, after sunset when the sun descends between 0° and –18° below the horizon [51]. In contrast, *M. midas* is nocturnal, with outbound foragers leaving the nest 20–40 min after sunset, during evening twilight. These foragers travel along the ground to one of the surrounding trees and climb up into the canopy to feed overnight [44]. Inbound navigation in *M. midas* is more variable, with foragers descending their foraging tree and returning to the nest entrance both overnight and into morning twilight (rising sun is between –18° and 0°) with activity ceasing by sunrise [44]. Both species are known to rely heavily on visual terrestrial cues while also accumulating a celestial-compass-derived path integrator for navigation (*M. tarsata*: [57]; *M. midas*: [7,44,45,48]).

### 2.2. Linear polarizer apparatus and testing procedure

During testing, the overhead polarization pattern was altered by placing a 30 cm diameter linear polarization filter [40] (Polaroid HN22) above individual foragers as they navigated back to their nest. This filter blocks the ambient e-vector pattern of the night sky, replacing it with the e-vector orientation of the filter, ± 45° to the ambient pattern (figure 1A). To allow foragers to travel beneath this filter, the linear polarizing film was held in place by a rigid holding ring raised 10 cm off the ground using four legs. Solar testing was conducted during evening or morning solar twilight, coinciding with the natural inbound navigation of *M. tarsata* and *M. midas*, respectively (figure 1B), while each lunar-phase condition was tested overnight before solar twilight during a waxing moon (crescent, quarter and full). We obtained the sunrise and moonset positions as they reached the horizon based on Astronomical Almanac measurements (https://aa.usno.navy.mil/publications/asa).

Across all solar and lunar tests, held ants were released at their collection site and allowed to travel towards their nest for 50 cm before the filter was placed overhead, and all testing occurred within the first 3 m from release (figure 1C). Each ant was tested with both the ± 45° e-vector rotation on a single inbound trip with the order randomized (either (i) +45° then –45° or (ii) –45° then +45°). For the first test, we recorded four positions along the ant’s route, the site they were released, the ant’s position when the (±45°) filter was placed overhead, the ant’s position when they exited the filter ring and their path approximately 50 cm after exiting the filter (reorientation heading). After reorientation, each ant was tested with the mirror e-vector-rotation filter orientation placed overhead. For this second test, we recorded their pre-filter heading (50 cm), the position when the filter was placed, the exit position and their position approximately 50 cm after exiting the filter (reorientation heading). The ant was followed during these periods using a red light headlamp, and stakes were used to mark the sites, taking care not to disturb the ant’s homing behaviour. These positions produced each forager’s initial heading under a natural e-vector pattern, their shift under an artificially rotated pattern and their reorientation back to the natural pattern after exiting the filter (figure 1A). After testing, each forager was recollected and marked with white acrylic paint (Tamiya™) to prevent retesting.

#### 2.2.1. Solar polarized light

For solar-polarized-light testing, foragers from both species were collected at the base of a foraging tree. *Myrmecia tarsata* foragers were collected from the ground approximately 6 m from their nest during their afternoon foraging period (16.00–18.00), fed honey and held in a 10 cm diameter glass phial at their capture site until evening twilight testing (corresponding with the ending of their foraging activity). *Myrmecia midas* foragers were collected during evening twilight (the onset of their outbound foraging) at the base of their foraging tree (approx. 6 m from the nest), just as they were about to climb onto the trunk. These foragers were fed honey and held in identical phials at the base of their foraging tree overnight and tested during the pre-dawn morning twilight, when the rising sun reached 15° below the horizon, and testing ceased at sunrise.

#### 2.2.2. Lunar polarized light

Polarized-moonlight testing began just after the moon set (0°) and ended before the moon descended below –18°, just after the setting moon crossed below the horizon until it reached 15° below the horizon. All testing occurred during waxing lunar phases, ensuring that the moon was present in the night sky from the end of solar twilight until testing, to reduce effects of overnight lunar-cue gaps [40]. We tested both species under three waxing lunar phases, representing distinct amounts of lunar illumination: full (>85%), quarter (~50%) and crescent (~25%). For all three lunar conditions, ants were collected identically to that for solar testing but released overnight, during moonset, when only the lunar polarization pattern was present in the overhead sky. Given a true 100%-illumination full moon that sets during morning solar twilight, when solar polarization pattern cues would dominate, we tested during the two nights preceding the full moon when illumination was >85%, but when the moon was just below the horizon and did not overlap with solar twilight.

### 2.3. Statistical analysis

Data were analysed with circular statistics with Oriana Software [58–60]. Shift magnitudes under the filter were calculated by taking the difference between each forager’s initial heading and its heading as it exited the filter. Within-individual comparisons (initial heading versus filter exit heading and filter exit heading versus reorientation) were analysed using Moore’s paired tests. To compare shift magnitudes between −45° and +45°, both within a condition as well as any potential effect of experimental order, we mirrored the shifts (across the 0°–180° plane) in each −45° condition for comparison with unaltered +45° shift data. We then compared these sets using Moore’s paired tests [50]. Across-species differences in shift magnitude were analysed using Watson–Williams *F*-tests between identical solar and lunar conditions (solar, full, quarter, crescent). For these cross-species comparisons, as each individual forager had two shift data points, we averaged these (+45° shift data and the mirrored −45°), creating a mean shift magnitude for each individual for comparison using Watson–Williams *F*-tests.

## 3. Results

### 3.1. Solar polarized light: twilight testing

**Figure 2.**
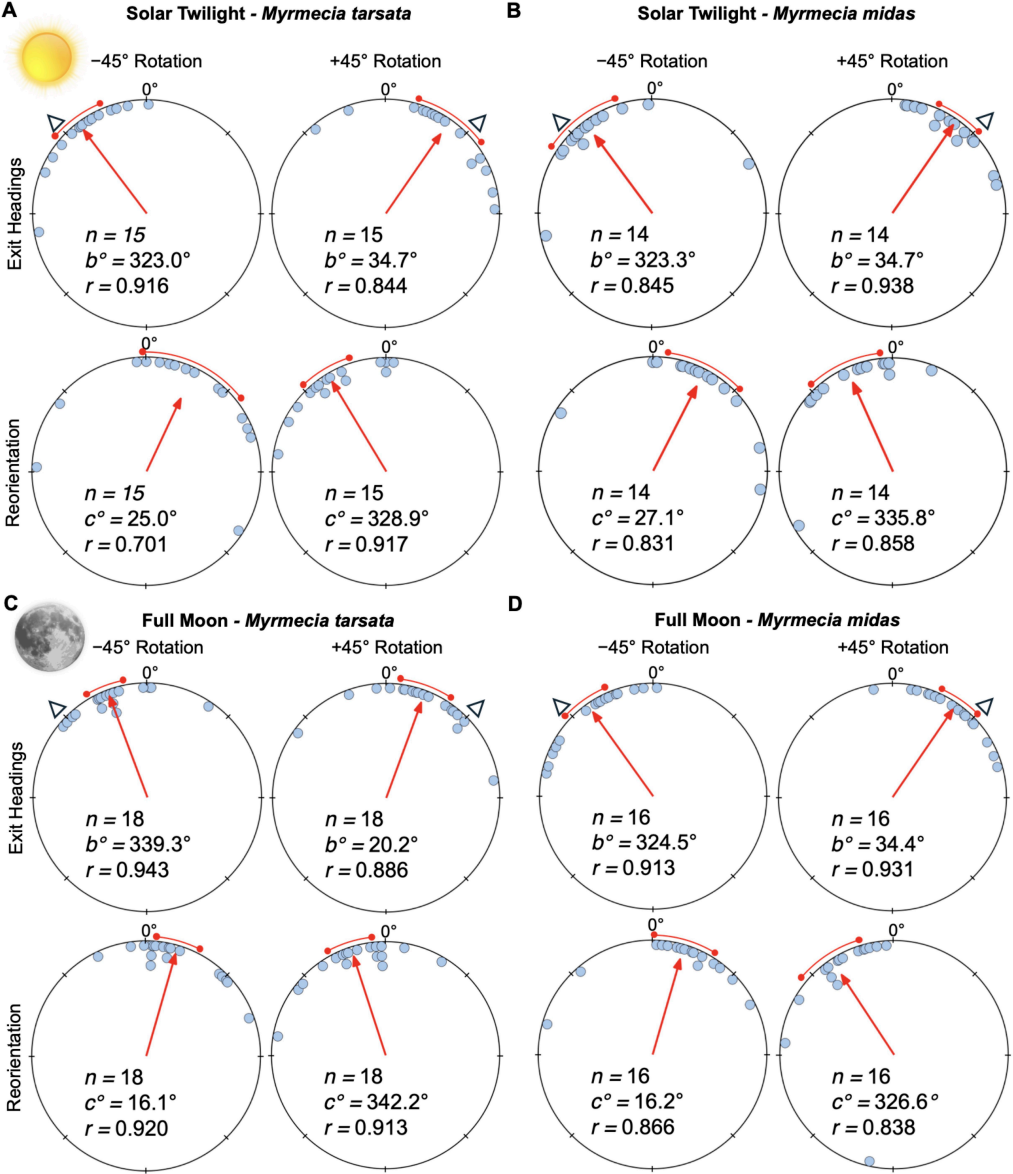
Circular distributions of headings during solar twilight and full moon conditions. In the solar condition, (A) *M. tarsata* and (B) *M. midas* were tested during their natural inbound twilight period, evening or morning twilight, respectively. Lunar testing in (C) *M. tarsata* and (D) *M. midas* occurred when the setting moon was just below the horizon (0° to –18°) and during the nights preceding the full moon (lunar illumination >85%). Plots show the exit headings of foragers relative to their initial headings, while the reorientation represents the change in headings 50 cm after exiting the filter. Triangles indicate the ±45° e-vector change when foragers are under the filter. Red arrows denote the length and direction of the mean vector of headings. *n*, number of individuals; *b*°, mean vector of shift; *c*°, mean vector of reorientation; *r*, length of the mean vector. Sun and moon images are public domain art accessed through wiki commons (https://commons.wikimedia.org/).

In both *M. tarsata* (evening twilight testing) and *M. midas* (morning twilight testing), when the linear polarized filter was rotated clockwise (+45°) of the ambient pattern and placed above inbound foragers, filter exit orientations were significantly shifted from each forager’s initial heading (table 1) predictably to the right of pre-filter headings (mean ± SE; *M. tarsata*: 34.7 ± 9.5°; *M. midas*: 34.7 ± 6.1°; figure 2A,B). Foragers also predictably altered their headings when the filter was positioned counterclockwise (–45°) of the ambient e-vector. Exit headings in both species were to the left of initial pre-filter headings (*M. tarsata*: –37.0 ± 6.9; *M. midas*: –36.7 ± 9.9°; figure 2A,B), and these shifts were significant (table 1).

**Table 1.** Shifts, reorientations and statistical comparisons for all tests. Negative degree values represent anticlockwise shifts, while positive values represent clockwise shifts.

	+45° shift			–45° shift			+45° reorientation			–45° reorientation		
	mean vector	Moore’s test		mean vector	Moore’s test		mean vector	Moore’s test		mean vector	Moore’s test	
		*R*′	*p*		*R*′	*p*		*R*′	*p*		*R*′	*p*
**solar twilight**												
*M. tarsata*	34.7°	1.496	<0.001	–37.0°	2.004	<0.001	–31.1°	1.6	<0.001	25.0°	1.343	<0.005
*M. midas*	34.7°	1.758	<0.001	–36.7°	1.483	<0.001	–24.2°	1.618	<0.001	27.1°	1.544	<0.001
**full moon**												
*M. tarsata*	20.2°	1.431	<0.005	–20.7°	1.089	<0.05	–17.8°	1.485	<0.005	16.1°	1.29	<0.01
*M. midas*	34.4°	1.783	<0.001	–35.5°	1.731	<0.001	–33.4°	1.902	<0.001	16.2°	1.115	<0.05
**quarter moon**												
*M. tarsata*	14.3	1.298	<0.01	–18.1°	1.59	<0.001	–19.2°	1.244	<0.025	22.8°	1.29	<0.01
*M. midas*	32.3	1.659	<0.001	–36.3°	1.604	<0.001	–26.6°	1.408	<0.005	33.5°	1.649	<0.001
**crescent moon**												
*M. tarsata*	3.8°	0.276	*0.791*	–4.0°	0.639	*0.393*	*–3.0°*	0.207	*0.836*	17.0°	0.643	*0.357*
*M. midas*	29.1°	*1.383*	*<0.005*	–33.0°	*1.558*	*<0.001*	*–23.1°*	*1.731*	*<0.001*	24.4°	*1.136*	*<0.05*

After exiting the (+45°) rotated filter, foragers of both species reoriented significantly (table 1) to the left (mean ± SE; *M. tarsata*: –31.1 ± 6.8°; *M. midas*: –24.2 ± 9.5°; figure 2A,B). A similar significant reorientation shift (table 1) to the right (mean ± SE; *M*. *tarsata*: 25.0 ± 13.8°; *M. midas*: 27.1 ± 6.8°) was observed in both species after exiting the (–45°) rotated filter.

### 3.2. Full moon lunar polarized light

Under a full moon (>85% illumination), when the polarization pattern was altered by +45° or –45° of the ambient pattern, foragers of both species significantly (table 1) altered their headings from pre-filter headings to the right (mean ± SE; *M. tarsata*: 20.2 ± 6.6°; *M. midas*: 34.4 ± 5.4°; figure 2C,D) or left, respectively (*M. tarsata*: –20.7 ± 5.5; *M. midas*: –35.5 ± 6.1°; figure 2C,D). In both species, after exiting the +45° or –45° rotated filter, foragers reoriented significantly (table 1) to the left (*M. tarsata*: –17.8 ± 5.8°; *M. midas:* –33.4 ± 8.4°; figure 2C,D) or right, respectively (*M. tarsata*: 16.1 ± 5.5°; *M. midas:* 16.2 ± 7.7°).

### 3.3. Quarter moon

**Figure 3.**
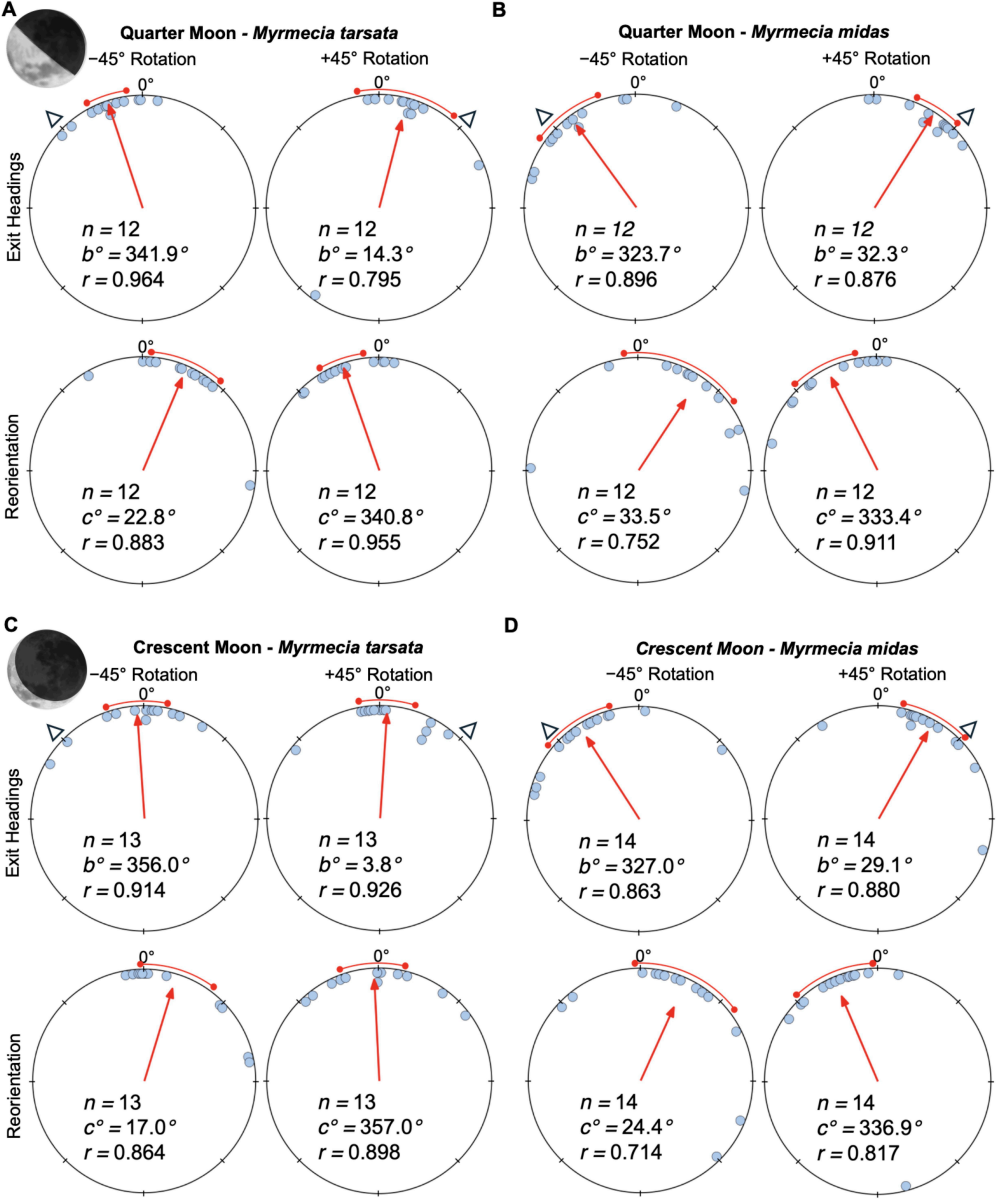
Circular distributions of headings during waxing quarter and crescent moon conditions. Under a quarter moon, (A) *M. tarsata* and (B) *M. midas* were tested when the moon’s illumination was approximately 50%. Crescent moon testing in (C) *M. tarsata* and (D) *M. midas* occurred when the moon was just below the horizon (0° to –18°) on nights when the moon phase was a waxing crescent (lunar illumination approx. 25%). Plots show the exit headings of foragers relative to their initial headings, while the reorientation represents the change in headings 50 cm after exiting the filter. Triangles indicate the ±45° e-vector change when foragers are under the filter. Red arrows denote the length and direction of the mean vector of headings. *n*, number of individuals; *b*°, mean vector of shift; *c*°, mean vector of reorientation; *r*, length of the mean vector. Moon images are public domain art accessed through wiki commons (https://commons.wikimedia.org/).

When tested under a waxing quarter moon and the overhead pattern was altered +45°, both species significantly (table 1; figure 3C,D) altered their headings (mean ± SE, *M. tarsata*: 14.3 ± 12.6°; *M. midas*: 32.3 ± 5.7°). Opposite significant shifts (table 1; figure 3C,D) to the left occurred when the overhead pattern was altered –45° (*M. tarsata*: –18.1 ± 5.1°; *M. midas*: –36.3 ± 8.4°). After exiting the filter, foragers reoriented significantly (table 1) to the left (*M. tarsata*: –19.2 ± 5.7°; *M. midas:* –26.6 ± 8.1°) or right, respectively (*M. tarsata*: 22.8 ± 9.4°; *M. midas:* 33.5 ± 7.7°).

### 3.4. Crescent moon

Under a waxing crescent moon, *M. tarsata* did not show significant shifts when the overhead pattern was altered ±45° (table 1; figure 3C). In contrast to *M. tarsata*, *M. midas* still attended to the updated polarized light pattern under the filter under a crescent (approx. 25% illumination) moon. When the overhead pattern was altered +45° or –45°, *M. midas* foragers significantly (table 1; figure 3D) altered their headings to the right or left, respectively (+45°, mean ± SE, 29.1 ± 12.1°; –45°, –33.0 ± 9.3°) and then significantly reoriented back to the ambient e-vector after leaving the filter (table 1; figure 3D).

### 3.5. Sequence and condition comparisons

Testing order within a homing trip between ±45° e-vector changes did not significantly alter shift magnitude within individuals in all conditions (Moore’s paired tests; *p >* 0.05). Additionally, shift magnitudes between ±45° conditions (within individual) did not significantly differ in either *M. tarsata* or *M. midas* (*p >* 0.05) across all conditions.

### 3.6. Across-species comparisons

As there were no differences between shift magnitudes, the two shifts for each individual were averaged, creating mean individual shifts for each solar and lunar condition. These mean individual shifts were used for cross-species comparisons.

*Solar* mean individual shift magnitudes under an altered solar polarized light pattern (*M. tarsata*: 35.7 ± 7.2°; *M. midas*: 35.5 ± 6.9°) were almost identical and not significantly different between species (Watson–Williams *F*-test; *F*_(1,27)_ = 0.001, *p* = 0.975), suggesting similar weighting for solar polarized light cues in both species.

Under full moon conditions, mean shift magnitudes were significantly different between species (Watson–Williams *F*-test; *F*_(1,32)_ = 6.154, *p* = 0.019), with *M. midas* exhibiting larger individual shift magnitudes (*M. tarsata*: 20.0 ± 3.3°; *M. midas*: 34.9 ± 4.9°). Under a quarter moon, *M. midas* again showed larger shifts (*F*_(1,22)_ = 5.240, *p* = 0.032; *M. tarsata*: 12.4 ± 8.4°; *M. midas*: 34.0 ± 6.1°). Finally, during a crescent moon, when *M. tarsata* did not show significant shifts, *M. midas* still maintained strong responses (*F*_(1,25)_ = 13.94, *p* < 0.001) during this lunar phase (*M. tarsata*: 4.2 ± 3.5°; *M. midas*: 30.6 ± 6.7°).

## 4. Discussion

### 4.1. Summary

These findings represent the second documented case of an insect using polarized moonlight for homing. In addition to the nocturnal *M. midas* [48], we now show that the diurnal/crepuscular *M. tarsata* also responds to lunar polarization patterns during path integration-based navigation. Both species reliably altered their headings in response to alterations of the ambient e-vector under a polarizing filter, demonstrating sensitivity to both solar [7] and lunar polarized light cues. Our results demonstrate that polarized light from both the sun and moon can be detected and integrated into the celestial compass of at least some diurnal or crepuscular insects. This raises the possibility that polarized moonlight sensitivity may be more common than previously recognized, even among species not typically active at night.

Under an altered solar polarization pattern during twilight, both *M. tarsata* and *M. midas* shifted their paths predictably, showing strong use of this cue. Under lunar polarization, *M. tarsata* responded significantly during full and quarter moons (though less than *M. midas*) but not under crescent conditions, indicating difficulty detecting the faintest patterns. By contrast, *M. midas* maintained strong shifts even under crescent moons, consistent with *M. midas*’ visual system being adapted for low-light detection.

### 4.2. Solar polarization pattern

Nocturnal bull ants (*M. midas* and *Myrmecia pyriformis*) are known to use the solar polarization pattern in the overhead sky to navigate during twilight periods [5,7,44]. Our results confirm that *M. midas* attends strongly to this cue, shifting 35.7° (79% of the 45° solar e-vector rotation), consistent with earlier findings [7].

Solar polarized-light use in *M. tarsata* has not previously been studied, but their inbound navigation stretching into twilight suggested they were also likely to rely on this cue. This prediction was supported as *M. tarsata* shifted 35.9° (80% of the filter rotation), similar to both *M. midas* and the diurnal/crepuscular desert harvester ant *Veromessor pergandei*, which also relies heavily on solar polarization cues after sunset [8]. These results indicate that this polarization cue is a robust and widespread compass cue across ant species navigating at twilight.

The consistent ‘underestimation’ of shifts (approx. 80–90% rather than a full 45°) across species is likely methodological. When the filter is placed overhead, heading shifts are not immediate as ants continue briefly in their original direction before turning. As the protocol measures the angle from the ant’s position at the filter centre when the filter is placed overhead rather than from the spot where the navigator altered its heading, measurements will inevitably slightly underestimate the shift [7,48].

### 4.3. Lunar polarization pattern

Many accounts of nocturnal *Myrmecia* suggest they restrict their navigation to outward trips during evening twilight and return trips in morning twilight [47], but in reality, they navigate extensively overnight when no solar cues are available [7,61]. Both *M. pyriformis* and *M. midas* therefore conduct true nocturnal navigation during these overnight hours. *Myrmecia tarsata*, although mostly diurnal, likely has some ability to detect faint polarized light given its twilight activity and reliance on solar cues after sunset. However, eye sensitivity studies [31,46,51,53] suggest it struggles under lunar patterns, particularly when only small portions of the moon are illuminated.

As expected, given when each species forages, *M. midas* attended remarkably well to the lunar e-vector throughout the lunar cycle, shifting approximately 77% of the filter rotation under full moons, approximately 76% under quarter moons and still approximately 69% under crescents. Additionally, in all conditions, the filter rotation fell within the 95% confidence interval of heading shifts (figure 2D; figure 3B,D), suggesting that *M. midas* foragers were fully attending to the updated polarization pattern. These heading shift values, only approximately 10% lower than under solar conditions, confirm that this nocturnal species can rely on lunar polarization cues across all phases, consistent with previous work [48].

By contrast, *M. tarsata* showed declining performance with decreasing lunar illumination: approximately 45% of the filter rotation under full moon, approximately 36% under quarter moon and no significant response under a crescent moon. Foragers often hesitated under the filter during crescent trials, suggesting the e-vector alone was below their detection threshold. Statistically, *M. tarsata* shifted significantly less than *M. midas* across all phases, indicating that while diurnal bull ants can detect lunar polarization, their sensitivity approaches threshold levels at lower lunar illuminations.

Despite these reductions, this represents only the second known case of a diurnal insect using polarized moonlight (after *S. lamarcki* [42]) and the second example of its use in goal-directed navigation (after *M. midas* [48]). Interestingly, mirroring the differences reported here, the diurnal dung beetle *S.* (*Kheper*) *lamarcki* also shows reduced performance under polarized moonlight compared to its nocturnal relative *S. satyrus* [42]. When the moon itself was visible, both species oriented equally well. However, when only the lunar polarization pattern (and stars) was available, diurnal beetles performed significantly worse, suggesting that dim-light eye adaptations are essential for accurate orientation under faint cues.

### 4.4. Eye morphology and visual adaptations

Differences in eye morphology between *M. tarsata* and nocturnal *Myrmecia* help explain why *M. tarsata* showed weaker responses to lunar polarization. Physiologically, the eyes of *M. tarsata* represent somewhat of a mid-point between fully diurnal bull ants such as *Myrmecia croslandi* and nocturnal bull ants *M. pyriformis* and *M. mida*s. Its facet lenses and photoreceptor diameters are larger than those of their diurnal counterparts (*M. croslandi*) yet smaller than those in the nocturnal *M. pyriformis* [51]. Both *M. tarsata* and *M. pyriformis* possess low-light compound-eye modifications, absent in diurnal bull ants [46]. In *M. tarsata*, proximal cone diameter doubles in the dark-adapted state, though this effect is less pronounced compared to *M. pyriformis* [46]. Comparisons with *M. midas* show that the latter has larger facets and higher contrast sensitivity, likely aiding navigation under very dim light. Such low-light adaptations enhance both landmark detection and celestial compass use at night [46]. Thus, while *M. tarsata* can readily detect the solar polarization pattern at twilight, its reduced visual sensitivity compared to nocturnal bull ants likely explains its declining performance under lunar polarization. In contrast, the stronger low-light adaptations of *M. midas* support reliable orientation across the lunar cycle [53,55,61] corresponding with its nocturnal activity schedule [38,44].

### 4.5. Pattern ambiguity

Despite the 180° ambiguous polarization pattern present during tests, foragers showed no evidence of becoming disoriented by this ambiguity, consistently orienting in the inbound direction of the e-vector. As all tests occurred during the same sun or moon elevations, the differences in shifts were thus the result of cue detection thresholds rather than directional uncertainty due to the linear e-vector. Given these ants are known to heavily weight view-based memories of their route over celestial information, it is likely that the presence of these cues (though partially occluded by the filter) helps resolve any ambiguity in the overhead pattern [44,62]. Additionally, the amount that the ambient e-evector was altered (45° instead of 90°) likely also aided foragers in choosing the correct direction under the filter using egocentric cues, making the least angular deviation from its pre-filter heading (45° instead of 135°).

### 4.6. Conclusions

The diurnal bull ant *M. tarsata* can detect and attend to both the solar polarized light pattern, present at twilight, as well as the faint polarized moonlight pattern to home to the nest overnight, though not throughout the full lunar cycle. Although *M. tarsata* performs similarly to the nocturnal bull ant *M. midas* when navigating under the solar polarization pattern present at twilight (when both species are naturally active), *M. tarsata* shows reduced homing performance under an altered lunar e-vector. This is reflected in smaller shift magnitudes compared to *M. midas*. This performance degrades further when lunar phase-associated illumination decreases, suggesting that *M. tarsata* struggles to detect or attend to the faint moonlight pattern of the quarter moon, while the pattern produced under a crescent moon may be too faint to be usable for navigation. In contrast, the nocturnal *M. midas* exhibits only small decreases in performance under even the faintest of polarization patterns produced under a crescent moon, suggesting the low-light-level visual adaptations possessed by nocturnal bull ants may be required for nocturnal navigation throughout the lunar cycle.

## Data Availability

All data, documentation and code are available at OSF [63].
